# Royal Westminster Ophthalmic Hospital

**Published:** 1830-01

**Authors:** 


### HOSPITAL REPORTS.

### ROYAL WESTMINSTER OPHTHALMIC HOSPITAL.

### *Report of Cases of Diseases of the Eyes.*

(Concluded from p. 524.)

Case VII. October 20th, 1829.—Peggy O'Donnell, æt. twenty-six, of sanguine temperament; appears in good health, with the exception of her eye. Reports that, about a month ago, she received a slight blow upon her left eye, which was immediately followed by an unpleasant and painful sensation. The pain increased, and redness of the anterior part of the eye ensued. She applied, of her own accord, four leeches one day, and two another day, and cold bread and water poultice. The leeches produced some relief, but the poultice none at all.

At present the eye appears irritable and considerably inflamed. The vessels of the conjunctiva covering the sclerotic are so much gorged with blood, as to give it the appearance of a dark pink patch. The cornea is muddy, and at its superior portion there is a pink patch, about the size of the white of the thumb-nail, having in its centre a few pustules. Vision is very obscure; can merely distinguish the shade of bodies. Has some pain in the eye during the day, but feels it very acute when she becomes warm in bed.—Habeat Sp. Terebinth. ʒi. ter de die.

22d.—Inflammation less; feels the eye improved and more comfortable.—Cont. medicam.

24th.—Improving. Cont. medicam.

25th.—Cont. Sp. Terebinth.

27th.—Can see a little better. Cornea more transparent; vessels less distended with blood; eye irritable.—Cont. medicament. Applic. vesicat. pone aurem sinistram. Pone in occul. Vin. Opii gtt. ij.

31st.—Repetatur Sp. Terebinth.

November 3d.—Considerably improved. Inflammation and irritation very much decreased.—Cont. medicam.

12th.—Cornea clearer; no pain of eye; sees and feels better. Medicine has produced a desire to make water more frequently.—Habeat Sp. Terebinth. ʒi. bis de die; et Decoct. Hord. c Pulv. G. Arab. et Sacch. ad libitum.

15th and 17th.—Continue.

19th.—Cornea almost clear, sight very much improved; no pain or uneasiness. Feels no irritation in the urinary organs; pulse regular; conjunctiva natural.

21st.—Cured.

Case by Richard Tuthill, M.D. Assistant Surgeon 52d Light Infantry.

Case VIII.—Sarah Strickland, æt. twenty-eight; admitted October 22d, 1829, with interstitial abscess of the cornea.

A large interstitial abscess at the lower and inner part of the cornea of the right eye; anterior chamber cloudy; high sclerotic inflammation; great pain in the ball of the eye and forehead; vision very defective. The inflammation has existed about a week. Does not know any cause.—Cucurb. cruent. ad ℥xij. de temp.—℞. Antim. Tartar. gr. ij.; Magn. Sulph. ℥ij.; Aq. fervent. ℔ss. Dissolve; sumatur 8vam partem omni horâ, si non excitat nauseam, antiquam dosem tertiam, sumatur doses duas omni horâ.

23d.—The cupping relieved the pain. The medicine did not excite nausea.—℞. Pulv. Ipecac. gr. xv.; Antim. Tart. gr. ij. M. fiat emeticus statim sumend.

24th.—Emetic operated well. Complains of a severe darting pain in the eye and brow, returning several times a day. Abscess appears ready to point; sclerotic inflammation is on the decline. Says she sees a little better.—Cucurb. cruent. ad ℥xij. ℞. Antim. Tartar. gr. ij.; Magn. Sulph. ℥i.; Aq. fervent. ℔ss. Solve, fiat mist. ut antea sumend.

25th.—Pain is not so severe, neither does it occur so frequently. Medicine caused a slight degree of nausea.—Repet. medicamenta.

26th.—The pain is not so violent; thinks she sees clearer. The collection of matter is evidently disappearing. Medicine does not nauseate, but it purges.—Cont. medicam.

27th.—Medicine does not nauseate, though it causes purging, and makes her feel very faint. Much freer from pain; can bear the light more easily; thinks she sees better.—Cont. medicament.; sumatur 8vam partem omni horâ et semisse.

28th.—Medicine does not nauseate. Complains of pain in the eye and head during the night. Vessels of the sclerotica are more injected, and much more numerous, of a reddish pink colour; great lachrymation, and intolerance of light. The collection of matter appears stationary.—Cucurb. cruent. ad ℥viij. de temp.—℞. Antim. Tartar. gr. iij.; Magn. Sulph. ℥i.; Aq. fervent. ℔ss. Solve, et sumatur 8vam partem omni horâ et semisse.

29th.—Free from pain; is a great deal better. The matter is being rapidly absorbed. Medicine excited considerable nausea this morning.—Cont. Antim. Tart.

30th.—Medicine continues to nauseate. Eye feels stronger.—Repet. medicam.

31st.—Repet. medicam.

November 1st and 3d.—Repet.

5th.—Is entirely free from pain, and altogether better; eye feels stronger, and she sees better. Matter is being fast absorbed. Sclerotic inflammation has nearly disappeared. The inflammation of the lids still exists, but is not so severe. Medicine continues to keep up a state of nausea and faintness, as well as purging, though the Antim. Tart. has been diminished for the last two days to two grains.—Rep. med. sum. 8vam partem 2dis horis.

7th.—Sees nearly as well as ever. There is a speck left in the place in the abscess.—Hydr. Subm. gr. vi. h. s. s.—Magn. Sulph. ℥i. mane.

10th.—Relapse. Sclerotic inflammation has returned in a severe degree; lids even more inflamed; cannot open her eye; vision very imperfect.—Appl. Ung. Argent. Nitr.

12th.—Says that the pain excited by the ointment was very severe. Can see much better, and open her eye easily; free from pain; sclerotic inflammation is much relieved; the inflammation of the lids exists in a high degree; speck appears rather less.—App. Ung. Arg. Nit. Hyd. Subm. gr. iv. omni nocte.

14th.—Is a great deal better; sclerotic inflammation has disappeared.—App. Ung. Arg. Nitr. Inf. Sennæ mane.

17th.—Nearly well; speck has nearly gone.—Ung. rep.

19th.—Returned the letter. To use the Gutt. Arg. Nitr.

Case kept by Mr. Foote.

